# PM2.5 exacerbates house dust mite-induced allergic rhinitis via STING-mediated nasal epithelial barrier disruption

**DOI:** 10.3389/fimmu.2026.1752415

**Published:** 2026-03-24

**Authors:** Youwei Bao, Zhiqiang Zhang, Qi Chen, Shi Binbin, Xinhua Zhu

**Affiliations:** 1 Department of Otorhinolaryngology Head and Neck Surgery, The Second Affiliated Hospital of Nanchang University, Nanchang, Jiangxi, China; 2 Jiangxi Medical College, Nanchang University, Nanchang, Jiangxi, China

**Keywords:** air pollution, allergic airway disease, environmental health, epithelial barrier, innate immunity, particulate matter

## Abstract

**Background:**

The synergistic effect of PM2.5 and house dust mite (HDM) in exacerbating allergic rhinitis (AR) is recognized, but the underlying molecular mechanisms remain unclear.

**Objective:**

This study investigated whether PM2.5 aggravates HDM-induced AR and nasal epithelial barrier damage via the STING signaling pathway.

**Methods:**

This research combined bioinformatics analysis of a human nasal transcriptome dataset with *in vivo* (BALB/c mouse model) and *in vitro* (Human Nasal Epithelial Cells, HNEpCs) experiments. Models were exposed to PM2.5 and HDM, alone or in combination. Rhinitis symptoms, epithelial barrier integrity, Th2 inflammation, and the STING/NF-κB pathway were assessed. The STING inhibitor H-151 was used for functional validation.

**Results:**

Bioinformatics analysis linked PM2.5 exposure to TNF/NF-κB signaling. *In vivo* and *in vitro* experiments consistently demonstrated that co-exposure to PM2.5 and HDM synergistically worsened nasal symptoms, Th2 responses (elevated IL-4, IL-5, IL-13, IgE), and impaired barrier function (downregulated E-cadherin and Claudin-1), while activating the STING/NF-κB pathway. Critically, H-151 treatment reversed these pathological changes.

**Conclusion:**

PM2.5 disrupts the nasal epithelial barrier and synergizes with HDM to exacerbate allergic inflammation by activating the STING/NF-κB pathway. This study identifies STING as a potential therapeutic target for environment-aggravated allergic diseases.

## 1 Introduction

Allergic rhinitis (AR) is an IgE-mediated inflammatory disorder of the nasal mucosa, affecting approximately 10%–30% of adults and up to 40% of children worldwide, posing a substantial burden on patients’ quality of life and socioeconomic systems (1, 2). Among various allergens, the house dust mite (HDM) is recognized as one of the most prevalent and major indoor allergens. Its sensitizing components (Der p 1, Der p 2) can trigger typical AR symptoms by activating a type 2 immune response (3).

In recent years, with the acceleration of global industrialization, atmospheric particulate matter (PM) pollution, particularly fine PM (PM2.5) and inhalable PM (PM10), has become a severe public health concern. Epidemiological studies have established that exposure to PM2.5 is not only an independent risk factor for respiratory diseases but also positively correlates with the incidence and severity of allergic conditions. Notably, PM2.5 acts beyond a mere carrier of allergens. As an environmental stressor, it can compromise the physical barrier of the nasal mucosal epithelium, increase allergen permeability—directly influencing the magnitude of the subsequent humoral immune response—and may create a pro-inflammatory microenvironment by inducing oxidative stress and innate immune activation. This process effectively “adjuvates” and amplifies the immune response to subsequent allergen exposure. However, the precise molecular mechanisms underlying the synergistic interaction between PM2.5 and HDM, particularly whether they involve specific intracellular signaling pathways, remain incompletely elucidated.

Stimulator of Interferon Genes (STING) is a central signaling molecule in the innate immune system, traditionally known for sensing cytosolic double-stranded DNA and initiating a type I interferon response against pathogenic infections. Emerging evidence, however, suggests a pivotal role for the STING pathway in sterile inflammation and chronic airway diseases. Specifically, aberrant STING expression has been observed in models of eosinophilic chronic rhinosinusitis with nasal polyps (eCRSwNP) and asthma, where it reportedly exhibits a negative correlation with Th2 inflammation levels (4–7). This implies that STING may possess novel functions in maintaining airway immune homeostasis beyond its classical role in anti-pathogen defense. Epidemiological and experimental studies have demonstrated that PM2.5 can act on nasal epithelial cells and local immune cells, triggering inflammatory responses through various pattern recognition receptors and signaling sensors. Specifically, PM2.5 can activate the MyD88-dependent NF-κB signaling pathway by being recognized by Toll-like receptors (such as TLR2 and TLR4) through lipopolysaccharides and polysaccharide components (8, 9). By inducing mitochondrial damage and the release of cytosolic DNA, it activates the cGAS-STING signaling axis, initiating type I interferon responses (10–12). Concurrently, PM2.5 can activate the NLRP3 inflammasome, promoting the maturation and release of IL-1β and IL-18 (13, 14). Furthermore, PM2.5-induced oxidative stress regulates the expression of antioxidant genes through the Nrf2/ARE pathway, while excessive oxidative damage directly activates the NF-κB pathway, promoting the production of pro-inflammatory cytokines (15). In nasal epithelial cells, PM2.5 exposure can also induce the release of epithelial-derived alarmins (such as IL-33, TSLP, and IL-25), which can directly activate type 2 innate lymphoid cells, creating a pro-Th2 microenvironment (16). Notably, the activation of these signaling networks by PM2.5 is dose- and time-dependent and may progressively disrupt epithelial barrier function through a “multiple-hit” mechanism, creating conditions for allergen penetration and immune activation. We therefore hypothesize that the STING pathway could be an under-explored bridge connecting environmental pollutant exposure and allergic airway inflammation (5, 17–19).

While allergic rhinitis has traditionally been viewed as a Th2-dominant inflammatory disease, the critical aspect of respiratory epithelial damage has often been overlooked. The integrity of the epithelial barrier directly determines the dose of allergen exposure and the extent of subsequent humoral immunity. Based on this background, we propose the scientific hypothesis that PM2.5 may disrupt nasal mucosal epithelial barrier integrity by aberrantly activating the STING signaling pathway in nasal epithelial cells, thereby synergizing with HDM to ultimately exacerbate the Th2-inflammatory response in AR (20–23). To test this hypothesis, this study integrated bioinformatics analysis, *in vivo* animal experiments, and *in vitro* cellular models to investigate whether PM2.5 exacerbates HDM-induced AR and epithelial damage via the STING pathway, providing a theoretical basis for developing preventive and therapeutic strategies against environmental-related allergic diseases.

## 2 Materials and methods

### 2.1 Data source and processing

The dataset for this study was sourced from the GSE215411 dataset (platform: GPL24676) in the GEO database, comprising FPKM transcriptome data of human nasal mucosal fibroblasts from three control groups and three PM2.5-exposed groups (total 50,464 mRNA samples). Differential expression analysis was performed using the limma package in R (version 4.1.2). After normalization, genes with |log2 Fold Change|> 1 and adj.P.Val <0.05 were identified as differentially expressed genes (DEGs), resulting in 345 significantly differentially expressed genes. These were visualized using ggplot2’s volcano plots. Subsequently, clusterProfiler was employed to conduct GO functional and KEGG pathway enrichment analyses (P <0.05 considered significant), with results presented through dotplots and barplots.

### 2.2 Material

PM2.5 (#SRM 2786) was obtained from the NIST (Gaithersburg, USA). The HDM (#XPB91D3A2.5) extract was supplied by the American laboratory Lenoir. The Imject™ alum adjuvant was provided by Thermo Fisher Scientific (Bremen). The sting inhibitor H-151 were supplied by MCE Company.

### 2.3 Animals and treatment protocols

Female BALB/c mice aged 6-8 weeks were provided by the Department of Animal Science at Nanchang University School of Medicine. The mice were housed under specific pathogen-free conditions at the Animal Center of Jiangxi Medical College, Nanchang University. Each group contained 6 female BALB/c mice aged 6-8 weeks. For the AR group, 200 μl sensitizing solution was administered via intraperitoneal injection on days 0, 7, and 14, followed by nasal administration of 20 μl triggering solution from days 21 to 27, once daily. The normal control group received equivalent PBS treatment and samples were collected on day 28.In order to reduce the influence of sex on immune response, this study uniformly selected female BALB/c mice, whose Th2 response is relatively stable (24, 25). All the animal experiments were complied with the guidelines of the Tianjin Medical Experimental Animal Care, and animal protocols were approved by the Institutional Animal Care and Use Committee of Yi Shengyuan Gene Technology(Tianjin)Co., Ltd.(protocol number YSY-DWLL-2024733), complying with global laboratory animal ethics guidelines.

sensitizing solution: Dissolve HDM dry powder in PBS to achieve a concentration of 1 mg/ml. Mix an appropriate amount with the 10-fold diluted aluminum hydroxide suspension at a 1:1 volume ratio. Administer 200 μl sensitizing solution per mouse via intraperitoneal injection. Stimulating solution: Prepare HDM nasal stimulant using PBS, containing 20 μg HDM per 20 μl PBS.

PM2.5 preparation: PM2.5 standard was provided as lyophilized powder (100 mg/vial). For cell experiments, PM2.5 suspensions at concentrations of 0, 25, 50, and 100 μg/ml were prepared in serum-free MEM medium within a sterile laminar flow hood. The suspensions were sonicated to ensure uniform dispersion and stored at -20 °C. For animal experiments, the required amounts of PM2.5 standard and sterile PBS were calculated. PM2.5 suspension (20 μl per mouse) was prepared using sterile PBS at a concentration yielding 100 μg PM2.5 per 20 μl. The suspension was sonicated during preparation, stored at -20 °C, and administered via intranasal instillation daily from day 21 to day 27.

#### 2.3.1 Characterization and contamination control of SRM 2786

Source and composition:NIST SRM 2786 is a certified reference material representing fine atmospheric particulate matter, with characterized levels of polycyclic aromatic hydrocarbons (PAHs), polychlorinated biphenyls (PCBs), and trace elements. Detailed compositional data are publicly available from NIST.

Biological contamination: All PM2.5 suspensions were prepared under sterile conditions, and routine mycoplasma testing of treated cells was negative throughout the study period.

### 2.4 Cell culture and processing

Human nasal epithelial cells (HNEpC) were provided by PromoCell Heidelberg (Germany). The cells were cultured at 37°C in an atmosphere of 5%CO2, and the medium was changed regularly as well as the proliferation and subculture of the cells. An *in vitro* model of the nasal epithelial barrier was also developed in which HNEpC under optimal growth conditions were seeded at a density of 2 × 105 cells into a 12-mm Transwell culture dish coated with 0.4 μm pore size polyester membrane. Cells were then further cultured and submerged in PneumaCult™-Ex Plus medium. Then, the medium was aspirated and added to PneumaCult™-ALI medium in the basolateral compartment, allowing air exposure to the apical compartment. Medium changes were performed every 2 days. During the drug intervention, HNEpC was treated with dimethyl sulfoxide (DMSO), sting agonist H-151 (1 μM, MCE, HY-112693), and diABZI STING agonist-1 (0.05 μM, MCE, HY-112693) for 24 hours (26, 27). Due to minor variations in cell passage numbers, ALI culture days, and intervention duration, absolute values may fluctuate between batches. However, the trend of key indicators and statistical significance remain consistent across all batches.

### 2.5 Statistics of nasal symptoms

In each group, symptoms, sneezing and scratching were recorded for 10 minutes after the last nasal stimulation on day 28.

### 2.6 Studies in organizational science

For histological analysis, the mouse head was decapitated, and the nasal septum was completely removed. The nasal mucosa was scraped using hook forceps and placed in EP tubes containing 10% neutral formalin for pathological staining. The lungs were fixed in 4% polyformaldehyde solution for 24 hours. The preserved nasal and lung tissues were embedded in paraffin, with 4mm-thick sections mounted on slides and dewaxed. HE and Masson staining were performed, followed by microscopic examination (400x magnification) using a Thermo Fisher Scientific microscope to analyze histopathological changes. HE staining was employed to assess inflammation in the nasal and pulmonary tissues. The degree of inflammatory cell infiltration around microairways was quantified using a 0-4 scoring system: 0-no cells; 1-minimal periluminal inflammation; 2-1-2 layers of inflammation; 3-3-5 layers; 4-over 5 layers. Masson staining evaluated collagen fiber deposition in airways, stained blue, with collagen fiber area percentages calculated using ImageJ software. PAS staining assessed glycogen content in nasal mucosa and bronchial lining, with red signals representing high-intensity areas, and percentage of red signal areas calculated using ImageJ software.

### 2.7 Immunohistochemical analysis

Immunohistochemistry was employed to determine the localization and concentration of sting protein. The lung tissue was sectioned in paraffin, dehydrated, rehydrated, and treated with 3% hydrogen peroxide. Sections were then blocked with 5% goat serum to minimize interference from non-specific immunoglobulins. The specimens were incubated at 37 °C for 2 hours with anti-sting antibody (Proteintech, product number 66680-1-Ig, dilution 1:500), followed by 30 minutes of incubation with secondary antibody at 37 °C. Protein expression was quantified using Image-J software, with magnification observed at 400x.The fluorescence intensity of immunofluorescence staining was quantitatively analyzed using ImageJ software. For each sample, five high-power fields (400×) were randomly selected to measure the average fluorescence intensity of E-cadherin or STING, and the mean was calculated after subtracting the background fluorescence. The data were expressed as relative fluorescence intensity (normalized to the control group). Cell counts were performed using DAPI staining.

### 2.8 ELISA

The ELISA kit (Wuhan Experimental Technology Co., Ltd., China) was used to detect the levels of IL-4 (meimian, MM-0165M1), IL-5 (meimian, MM-0164M1), IL-13 (meimian, MM-0173M1), and total IgE (meimian, MM-0056M1) according to the manufacturer’s instructions. In order to detect IL-4, IL-5, IL-13 and IgE, 50 μL of mouse serum samples were added into each well. All the operations were carried out in strict accordance with the instructions of the ELISA kit.

### 2.9 Western blotting

Protein was extracted from lung tissue using RIPA lysis buffer containing protease inhibitors (GRF101, Epizyme) and a phosphatase inhibitor mixture (GRF102, Epizyme), with quantification performed using the BCA protein detection kit (ZJ101, Epizyme). Equally loaded proteins were electrophoretically separated on polyacrylamide gel and transferred to polyvinylidene fluoride (PVDF) membranes. Primary antibodies incubated at room temperature for 2 hours included: actin (ProteTech, 60008-1-Ig, 1:5000), sting (ProteTech, 66680-1-Ig, 1:5000), P-NF-KB (CST, 3033T,1:1000), and NF-KB (CST, 8242T,1:1000),E-cadherin(ProteTech, 20874-1-AP,1:20000),Claudin-1(ProteTech,28674-1-AP,1:1000). After 40 minutes of secondary antibody incubation at room temperature, cell membrane detection was performed using a chemiluminescent substrate system (Bio-Rad Laboratories, CA, USA). Protein band intensity was quantitatively analyzed using Image J software. ECL luminescence solution from Huch/Wilber company was used for development.

### 2.10 Cell viability assessment

To evaluate the percentage of viable cells using the CCK-8 assay kit: Briefly, well-grown HNEpC cells were inoculated into 96-well plates and exposed to HDM (0, 50,100,200,300μg/mL) for 24 hours. After drug removal, microscopic examination was performed on each well to observe cell morphology. Each well received 10 μL CCK-8 detection reagent, followed by incubation at 37°C for 2 hours. The results were quantified using a microplate reader (Thermo Fisher Scientific, Germany) to measure absorbance at 450 nm, facilitating evaluation of cellular viability.

### 2.12 Assessment of epithelial barrier integrity

The assessment of HNEpC epithelial barrier was conducted using transepithelial resistance (TEER) and fluorescein-difluoropropylglucoside (FD4) permeability assays. Use the Millipore Millicell ERS-2 ohmmeter and calibrate the Transwell blank before each measurement. The electrodes of an ohmmeter were placed in both the top chamber and bottom chamber of transwell inserts, with the instrument generating TEER values expressed in Ω·cm² (TEER measurement multiplied by membrane surface area). A TEER value ≥300 Ω·cm² indicates successful establishment of a cellular barrier. Following TEER measurements, HNEpC permeability was further evaluated. FD4 (800 μg/mL, Thermo) was added to the top chamber of transwell inserts for 2 hours, after which the fluorescence spectrophotometer (Thermo) was used to measure the concentration of FD4 in the supernatant of the bottom chamber.

### 2.11 Data analysis

The data were expressed as mean ± standard deviation (SD). All analyses were performed using GraphPad Prism^®^ 10.0 software. Independent samples t-tests and standard one-way ANOVA were used for pairwise and multiple group comparisons, respectively, with a significance threshold set at p <0.05. Tukey’s correction was applied for multiple comparisons.

## 3 Result

### 3.1 GEO data analysis reveals PM2.5-induced upregulation of TNF and NF-κB

**Figure 1:**
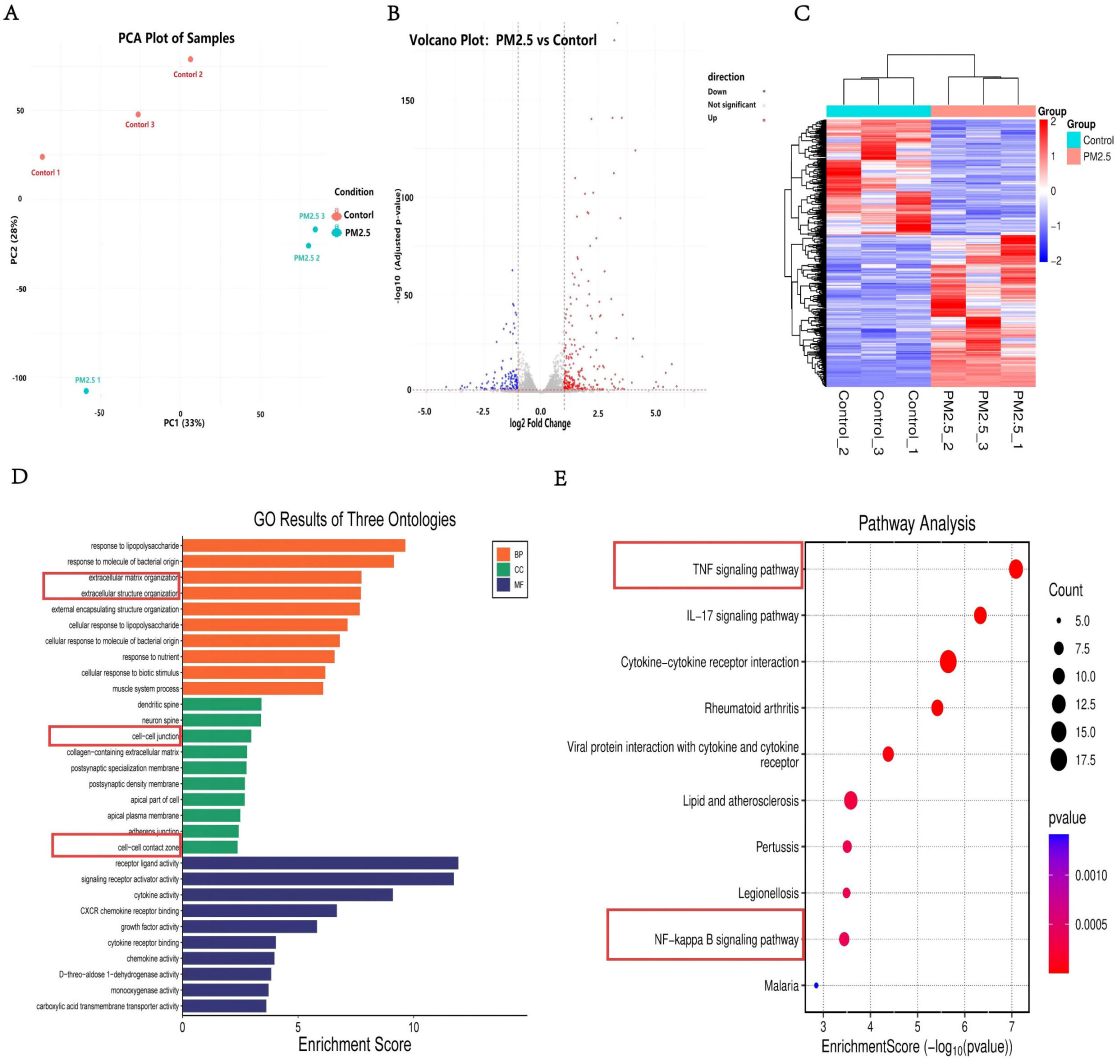
Bioinformatics analysis of transcriptome data. A.PCA Plot of Samples. **(B)** Volcano Plot: PM2.5 vs Control. **(C)** Top 50 Differentially Expressed Genes. **(D)** GO Results of Three Ontologies. **(E)** KEGG Pathway Enrichment Score dotplot.

To gain a global perspective on the transcriptomic impact of PM2.5 on nasal mucosal epithelial cells, we analyzed the GSE215411 dataset (Platform: GPL24676). Principal component analysis (PCA) revealed a clear separation trend between the PM2.5-treated group and the control group along the first principal component (PC1), which accounted for 33% of the total variance (**Figure 1A**). Samples within each group clustered well, indicating high reproducibility and consistency in the experimental procedures. This was further confirmed by a heatmap of inter-sample gene expression correlations, which showed high correlation coefficients (Pearson r > 0.98) within groups and significantly lower correlations between groups. Based on this high-quality transcriptomic data, we performed differential expression analysis using thresholds of |log2 Fold Change| > 1 and adjusted P-value (adj.P.Val) < 0.05. The volcano plot (**Figures 1B, C**) visually displays the global distribution of differentially expressed genes (DEGs): a total of 345 significant DEGs were identified, of which 223 were significantly upregulated and 122 were significantly downregulated. These widespread transcriptomic alterations suggest that PM2.5 exposure profoundly disrupts the gene expression profile in nasal mucosal epithelial cells. To elucidate the biological implications of these DEGs, we performed Gene Ontology (GO) functional enrichment analysis and Kyoto Encyclopedia of Genes and Genomes (KEGG) pathway enrichment analysis. The GO enrichment bubble plot (**Figure 1D**) showed that the DEGs were significantly enriched in biological processes closely related to inflammation and immune responses, such as “inflammatory response,” “extracellular matrix organization,” “response to biotic stimulus,” “cell-cell contact zone,” and “cell-cell junction.” This indicates that PM2.5 intervention can affect epithelial cell junctions. Crucial KEGG pathway analysis revealed a more systematic regulatory network. As shown in **Figure 1E**, the upregulated genes were significantly enriched in several classical pathways, including the “TNF signaling pathway,” “NF-kappa B signaling pathway,” and “cytokine-cytokine receptor interaction.” These enriched pathways are recognized downstream pathways of the STING signaling pathway.

### 3.2 Animal experiments reveal that PM2.5 synergistically exacerbates HDM-induced allergic rhinitis

**Figure 2:**
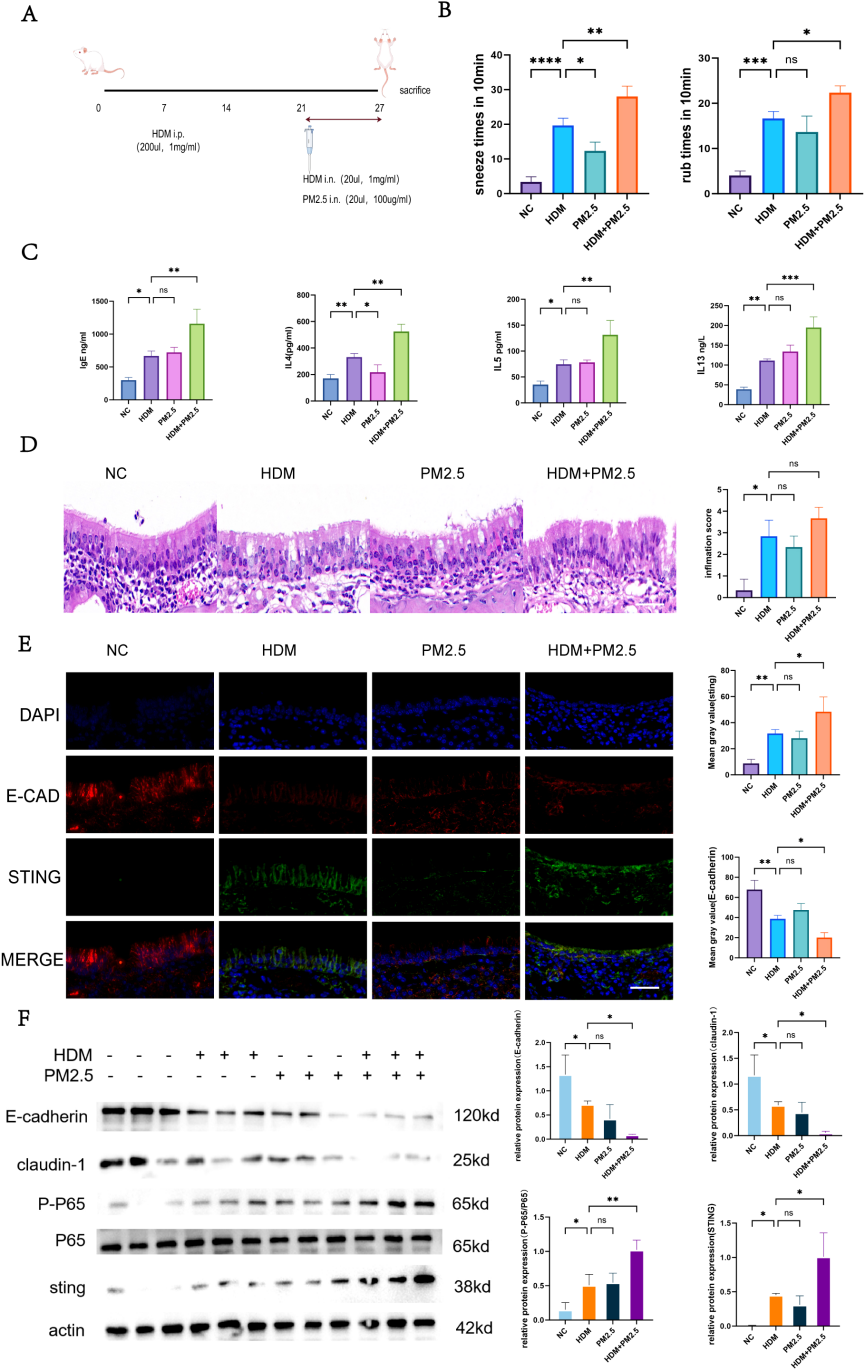
PM2.5 and HDM synergistically aggravate allergic rhinitis symptoms and epithelial damage in mice. **(A)** Schematic of animal model construction. **(B)** Behavioral statistics. **(C)** ELISA assay for serum IL-4, IL-5, IL-13, and IgE. **(D)** HE staining to assess nasal mucosal inflammation. **(E)** IF (E-cadherin, STING) staining for nasal mucosal signaling. **(F)** Western blot analysis of nasal mucosal protein levels, scale bars=50 µm. Ns indicates no statistically significant difference,*p < 0.05, **p < 0.01, ***p < 0.001, ****p < 0.0001 vs. indicated groups.

To investigate the impact of PM2.5 on house dust mite (HDM)-induced allergic rhinitis (AR), a BALB/c mouse model was established with the following experimental groups: normal control (NC), HDM alone sensitization group (HDM), PM2.5 alone exposure group (PM2.5), and HDM combined with PM2.5 co-exposure group (HDM+PM2.5). Behavioral observations indicated that both HDM and PM2.5 alone could induce typical AR symptoms, including a significant increase in nose scratching and sneezing frequencies; however, no statistically significant difference in symptom severity was observed between these two single-exposure groups. In contrast, mice in the HDM+PM2.5 co-exposure group exhibited significantly aggravated rhinitis symptoms compared to the HDM alone group (**Figures 2A, B**), suggesting a clear synergistic effect of PM2.5 in amplifying the sensitization effect of HDM.ELISA results showed comparable serum levels of Th2 cytokines (IL-4, IL-5, IL-13) and IgE in the HDM and PM2.5 groups, both being higher than those in the NC group, but with no significant difference between the two single-exposure groups. Notably, HDM+PM2.5 co-exposure significantly elevated the levels of these indicators compared to the HDM alone group, with statistical significance (**Figure 2C**), indicating that PM2.5 synergistically enhanced the Th2-polarized immune response with HDM. Histopathological analysis of nasal mucosal tissue further confirmed these findings. H&E staining revealed that compared to the HDM group, the HDM+PM2.5 group exhibited more severe tissue damage, characterized by extensive loss of nasal cilia, disruption of epithelial layer continuity, and significant thickening of the submucosa (**Figure 2D**). Immunofluorescence (IF) staining demonstrated a significant reduction in the signal intensity of the key epithelial barrier protein E-cadherin, alongside a marked increase in STING protein expression in the HDM+PM2.5 group (**Figure 2E**). To validate these phenotypes at the molecular level, Western Blot analysis was performed to detect the protein expression of epithelial tight junction proteins and inflammation-related signaling pathways in nasal mucosal tissues. The results showed that, compared to the HDM group, the HDM+PM2.5 group had significantly downregulated protein expression of E-cadherin and Claudin-1, while the protein levels of STING and the ratio of P-P65/P65 were significantly upregulated (**Figure 2F**). No significant differences in the expression of these proteins were found between the PM2.5 alone group and the HDM group.

### 3.3 *In vitro* HNEpC experiments reveal PM2.5 exacerbates cellular damage via the STING pathway

**Figure 3:**
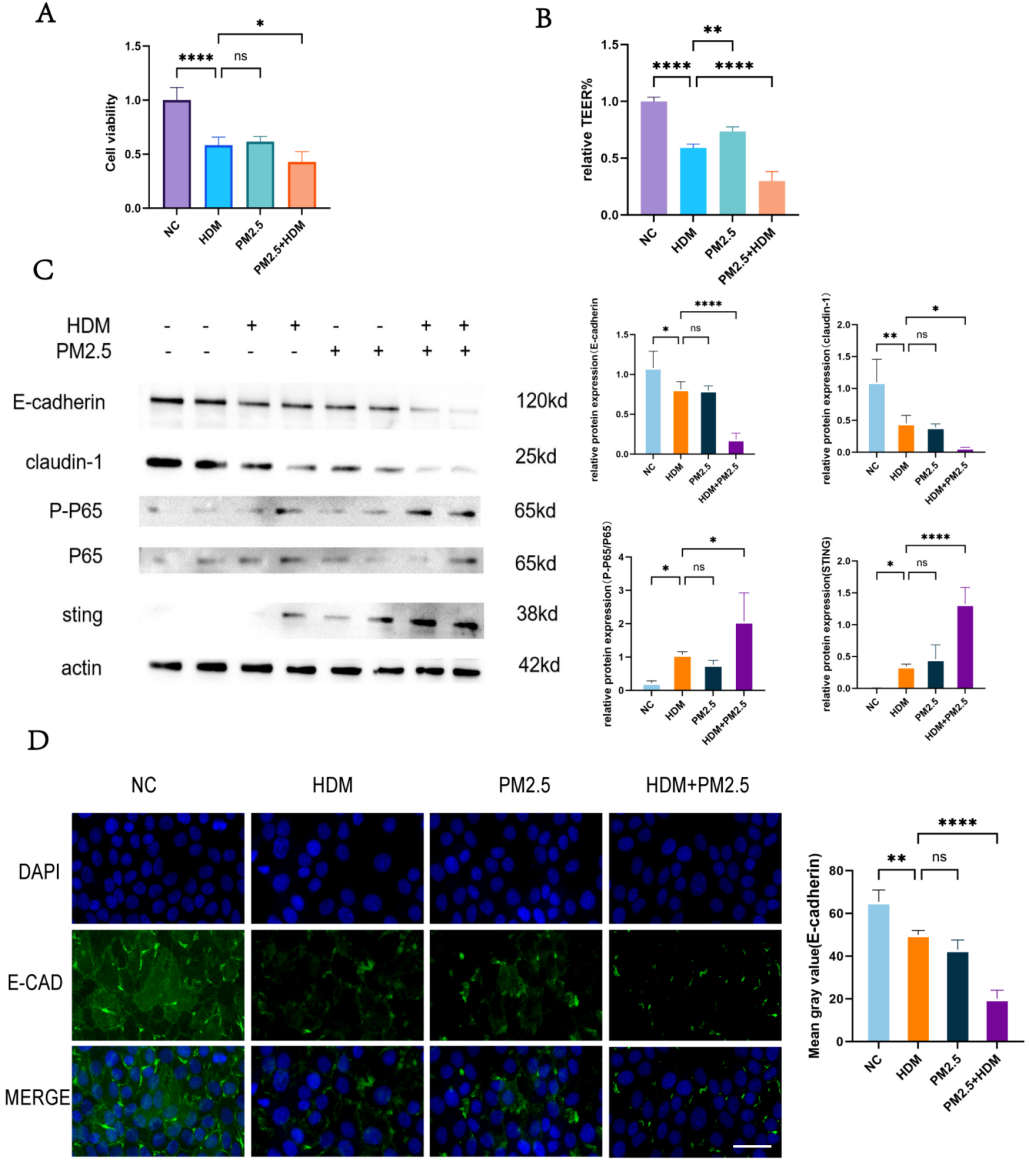
PM2.5-induced HDM-induced HNEpC damage. **(A)** CCK-8 assay for cell viability. **(B)** TEER assay for epithelial resistance. **(C)** WB assay for protein levels. **(D)** IF assay for signaling levels, scale bars=50 µm. Ns indicates no statistically significant difference,*p < 0.05, **p < 0.01, ****p < 0.0001 vs. indicated groups.

To validate the synergistic damaging effects of PM2.5 and HDM *in vitro*, experiments were conducted using Human Nasal Epithelial Cells (HNEpCs). The CCK-8 assay results demonstrated that combined HDM and PM2.5 treatment (HDM+PM2.5) significantly reduced cell viability compared to the HDM-alone group. The absorbance value in the co-exposure group was significantly lower than that in either the HDM or PM2.5 single-exposure groups (**Figure 3A**), indicating a synergistic inhibitory effect on cell viability. Assessment of epithelial barrier function yielded consistent findings. Measurement of the Trans-epithelial Electrical Resistance (TEER) revealed that the HDM+PM2.5 co-treatment induced the most pronounced decline in TEER value, which was substantially lower than that observed in any of the single-treatment groups (**Figure 3B**). This suggests that PM2.5 exacerbates the disruption of epithelial barrier integrity caused by HDM. To further investigate the molecular mechanism underlying this synergistic damage, we analyzed barrier proteins and related signaling pathways via Western Blot (WB) and Immunofluorescence (IF). WB results confirmed that, compared to HDM treatment alone, HDM+PM2.5 co-treatment significantly downregulated the expression of the epithelial tight junction proteins E-cadherin and Claudin-1, while simultaneously upregulating STING protein levels and the P-P65/P65 ratio (**Figures 3C, D**). No statistically significant differences in the expression levels of these proteins were observed between the HDM and PM2.5 single-exposure groups. Immunofluorescence staining corroborated these findings, showing the weakest E-cadherin fluorescence signal intensity and blurred cellular outlines in the HDM+PM2.5 group, providing further evidence of the most severe disruption to the epithelial barrier structure under this condition (**Figure 3E**). In summary, our *in vitro* experiments indicate that while PM2.5 alone has a limited effect, it significantly potentiates HDM-induced damage to nasal mucosal epithelium. The mechanism is likely associated with the synergistic downregulation of tight junction proteins and activation of the STING/NF-κB inflammatory signaling pathway.

### 3.4 Inhibition of the STING pathway mitigates PM2.5-induced damage in HNEpCs

**Figure 4:**
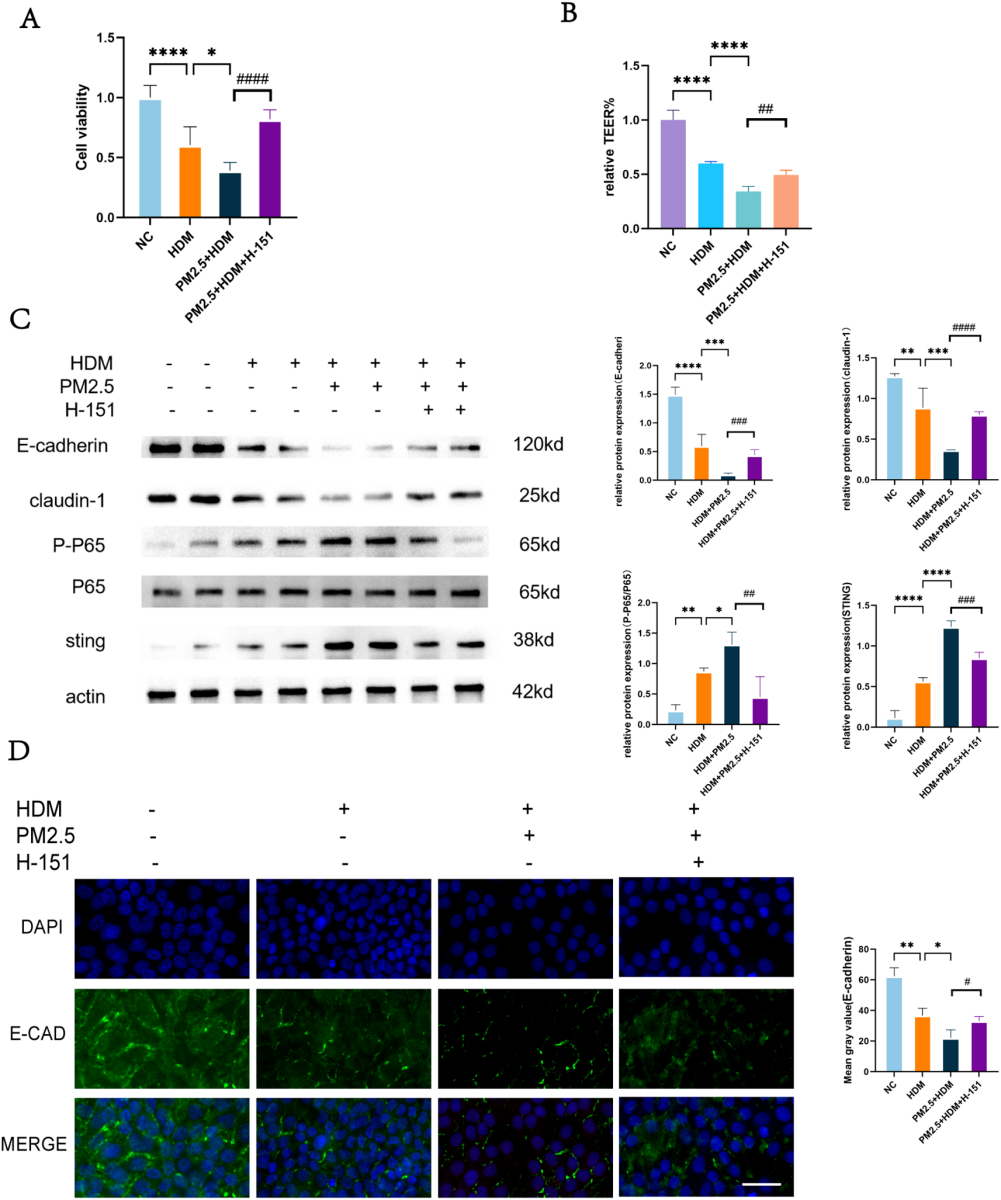
Intervention in the STING pathway of HNEpC cells improves epithelial injury. **(A)** CCK8 assay for cell viability. **(B)** TEER assay for epithelial resistance. **(C)** WB assay for cellular protein levels. **(D)** IF assay for cellular protein levels, scale bars=50 µm. Ns indicates no statistically significant difference,*p <0.05, **p < 0.01, ***p < 0.001, ****p < 0.0001,#p < 0.05 ^##^p < 0.01, ^###^p < 0.001, ^####^p < 0.0001 vs. indicated groups.

To elucidate the role of the STING pathway in the synergistic damage induced by PM2.5 and HDM, we intervened in Human Nasal Epithelial Cells (HNEpCs) using the specific STING inhibitor H-151. The results demonstrated a clear protective effect conferred by STING pathway inhibition. Under the damaging conditions of PM2.5+HDM co-exposure, the addition of H-151 led to a significant recovery of the suppressed cell viability (**Figure 4A**). Concurrently, the Trans-epithelial Electrical Resistance (TEER), an indicator of epithelial barrier integrity, also showed a significant increase (**Figure 4B**). This confirms that inhibiting the STING pathway effectively alleviates the decline in cell viability and the disruption of barrier function caused by combined PM2.5 and HDM exposure. At the molecular level, Western Blot analysis revealed that, compared to the PM2.5+HDM group, the H-151 intervention group exhibited significantly upregulated expression of the epithelial tight junction proteins E-cadherin and Claudin-1, while the activation level of the NF-κB pathway (indicated by the P-P65/P65 ratio) was significantly suppressed (**Figure 4C**). Immunofluorescence staining provided morphological evidence for these findings: the E-cadherin fluorescence signal, which was significantly weakened and disorganized in the PM2.5+HDM group, showed a marked trend of recovery after H-151 intervention, with cellular outlines becoming clearer and more continuous (**Figure 4D**). In summary, the STING inhibitor H-151 effectively reversed the series of adverse phenotypes triggered by the synergistic action of PM2.5 and HDM. These functional rescue results demonstrate that the overactivation of the STING pathway is a critical molecular event mediating the exacerbation of HDM-induced nasal mucosal epithelial damage by PM2.5 in the context of allergic rhinitis.

## 4 Discussion

Based on an integrated approach combining bioinformatics analysis, *in vivo* animal experiments, and *in vitro* cellular studies, this research systematically elucidates the molecular mechanism by which PM2.5 exacerbates allergic rhinitis—specifically, the Th2 inflammatory response and nasal mucosal epithelial barrier damage—through activation of the STING signaling pathway in synergy with HDM. The main findings of the study are as follows:Transcriptomic analysis indicated that PM_2.5_ exposure is closely associated with activation of the TNF/NF-κB signaling pathway downstream of STING. Animal experiments demonstrated that combined exposure to PM2.5 and HDM exhibited a significant synergistic effect, jointly aggravating rhinitis symptoms, Th2 immune responses, and nasal mucosal barrier injury. Experiments using HNEpCs revealed that this synergistic damaging effect was accompanied by activation of the STING/NF-κB pathway and downregulation of tight junction proteins. Pharmacological inhibition of the STING pathway with H-151 effectively reversed the aforementioned pathological processes. These findings provide new insights into how environmental pollutants exacerbate allergic airway diseases.

### 4.1 Synergistic effect of PM2.5 as an environmental stressor with allergens

This study found that in animal experiments, while exposure to either PM2.5 or HDM alone induced responses of comparable magnitude in terms of behavioral changes, pathological alterations, and the expression of key epithelial proteins (E-cadherin, Claudin-1) detected by Western Blot, their combined exposure demonstrated a significant synergistic amplification effect. Immunofluorescence detection of the mouse nasal mucosa revealed the lowest expression level of epithelial proteins in the PM2.5+HDM co-exposure group, with no statistically significant difference observed between the PM_2.5_ alone and HDM alone groups. In cellular experiments, co-exposure of Human Nasal Epithelial Cells (HNEpCs) to PM_2.5_ and HDM synergistically exacerbated cellular injury, specifically manifested as reduced cell viability capacity and decreased Transepithelial Electrical Resistance (TEER). Western Blot and Immunofluorescence results consistently confirmed the significant downregulation of epithelial barrier-related proteins, aligning with the findings from the animal studies. These results are consistent with a growing body of epidemiological observations suggesting that while air pollutant exposure itself may not directly initiate allergic sensitization, it can significantly exacerbate symptom severity and frequency in allergic individuals. The underlying synergistic mechanism may involve two aspects: Firstly, PM_2.5_, as an environmental stressor, compromises the physical barrier of the nasal mucosal epithelium (evidenced by downregulation of E-cadherin and Claudin-1), thereby enhancing the penetration of HDM across the epithelial barrier and increasing the cumulative allergenic exposure dose in sensitized individuals(T. 27–31). Secondly, as a complex mixture of atmospheric pollutants, certain components of PM2.5 may act as adjuvants, directly activating the local innate immune system and creating a pro-inflammatory microenvironment that amplifies the subsequent adaptive immune response triggered by HDM (32–35). The significantly higher serum levels of Th2 cytokines and IgE in the HDM+PM2.5 group compared to the HDM alone group in animal experiments strongly supports this perspective.

It is noteworthy that in this study, PM2.5 alone exhibited phenotypes similar to those of the HDM group in certain indicators (e.g., elevated Th2 cytokines, increased total IgE), but this does not imply identical molecular mechanisms. As a classical allergen, HDM activates specific Th2 immunity through antigen presentation, whereas PM2.5 induces nonspecific inflammation and polyclonal IgE production via barrier disruption, oxidative stress, and adjuvant effects. This “epigenetic similarity” precisely reflects the complex interactions between environmental pollutants and allergens—although PM2.5 cannot directly induce antigen-specific immunity, it creates a pro-inflammatory microenvironment that provides a “substrate” for allergen sensitization. Future studies may further differentiate their immunological characteristics through assays such as HDM-specific IgE detection and T-cell viability experiments.

### 4.2 The STING pathway: a novel link between PM2.5 exposure and nasal inflammation

The most salient finding of this study lies in revealing the central role of the STING signaling pathway in PM2.5-aggravated allergic rhinitis (AR). Both *in vivo* and *in vitro* experiments demonstrated that the protein expression of STING and the P-P65/P65 ratio were significantly enhanced under PM2.5 and HDM co-exposure. While traditionally studied predominantly in the context of tumor immunity for its role in responding to cytosolic pathogenic DNA, our findings indicate that the STING pathway can also be activated under sterile inflammatory conditions, specifically by synergistic PM2.5 and HDM exposure. We propose that PM2.5 might induce mitochondrial stress, leading to the release of host DNA, thereby mimicking a “pseudo-infection” state and triggering the innate immune alarm system centered on the cGAS-STING axis. This aligns with recent observations of STING activation in non-infectious airway inflammation. Furthermore, this mechanism corresponds with our previous research demonstrating that artesunate alleviates allergic rhinitis-related epithelial damage by modulating the STING pathway (4, 36). Both our bioinformatic analysis and experimental validation consistently pointed towards the activation of NF-κB downstream of STING, rather than the canonical Type I interferon response (5, 17, 18). This preferential signaling outcome may be determined by cell type, the nature of the stimulus, or signal intensity. The activation of the STING pathway directly led to NF-κB phosphorylation, which in turn drives the transcription of a cascade of pro-inflammatory factors and chemokines. This sequence is likely the direct cause of enhanced local inflammatory cell infiltration and the exacerbation of Th2 immune polarization. Following intervention with the STING-specific inhibitor H-151, the PM2.5+HDM co-treatment-induced reductions in cell viability, decreases in TEER, downregulation of tight junction proteins, and activation of NF-κB were all significantly ameliorated. This pharmacological blockade experiment provides functional evidence for the involvement of the STING pathway in this pathological process: inhibiting STING activity was sufficient to attenuate the synergistic damaging effects of PM2.5+HDM, suggesting that STING pathway activation plays a key role in this model. However, it must be interpreted cautiously, as pharmacological inhibitors may have off-target effects, and H-151 intervention only demonstrates the “necessity” of STING activity, without yet verifying its “sufficiency” through means such as gene knockout.

### 4.3 Therapeutic potential and perspectives of targeting the STING pathway

The cellular intervention experiments in this study provide direct evidence supporting the STING pathway as a therapeutic target. Treatment with the STING inhibitor H-151 restored nasal epithelial barrier function and significantly mitigated inflammatory responses. Specifically, this was demonstrated by the recovery of cell viability in CCK-8 assays, the restoration of transepithelial electrical resistance (TEER), and the normalized expression of key proteins as confirmed by Western Blot and immunofluorescence. These findings suggest that targeted inhibition of the STING pathway could be an effective strategy for alleviating pollution-associated allergic airway diseases. While activation of the STING pathway has been implicated in tumor immunity, its overactivation may lead to excessive cellular responses. Therefore, maintaining STING signaling homeostasis should be a key consideration in future research on epithelial immunity (J. S. 37, 38).

This study has several limitations. First, given the complex composition of PM2.5, the specific components (e.g., metals, polycyclic aromatic hydrocarbons) primarily responsible for driving STING activation remain unidentified. Second, the upstream activating signals of the STING pathway, particularly the source of cytosolic DNA, require further confirmation. Future research will focus on elucidating the precise upstream mechanisms by which PM_2.5_ triggers the STING pathway and validating these findings in more human-relevant models, such as human airway epithelial organoids. This study has certain limitations in establishing the causal role of the STING pathway. The current primary evidence includes: increased STING protein expression and elevated NF-κB phosphorylation levels in the PM2.5+HDM co-exposure group; the use of the STING-specific inhibitor H-151 partially reversing PM2.5+HDM-induced cellular damage and barrier disruption. Although the inhibitor experiment provides loss-of-function evidence supporting a key role for the STING pathway in this model, more rigorous causal validation methods, such as gene knockout or siRNA silencing, have not yet been employed. Therefore, the current evidence should be interpreted as indicating that STING pathway activation is closely associated with this pathological process, rather than having been established as a “necessary condition.” Future studies could employ STING knockout mice or STING-siRNA knockdown cells to further verify the causal role of STING in this synergistic effect.

In summary, by integrating bioinformatic analysis, animal models, and cellular experiments, this study confirms that PM2.5 exhibits a significant synergistic effect with house dust mite (HDM), collectively exacerbating the clinical symptoms of AR, Th2-type inflammation, and nasal mucosal epithelial barrier damage. Under certain exposure conditions, this synergistic damage exceeds the additive effects of individual factors. Mechanistically, our research reveals that aberrant activation of the cGAS-STING signaling pathway is a critical mediator of this synergy. The combined action of PM2.5 and HDM triggers the STING pathway, driving downstream NF-κB activation, which promotes the release of inflammatory factors and disrupts epithelial tight junction proteins (E-cadherin, Claudin-1), ultimately leading to nasal mucosal barrier dysfunction. These findings not only deepen the understanding of environment-gene interactions in allergic diseases but also provide a scientific foundation and new direction for developing STING-targeted interventions to mitigate the adverse public health impacts of air pollution.

## 5 Conclusion

This study not only proposes a novel mechanism of environmental exposure-induced sensitization through “PM2.5-STING-NF-κB-epithelial barrier damage,” but also provides groundbreaking experimental evidence for understanding the pathological processes of air pollution exacerbating allergic diseases. From the perspective of translational medicine, the STING signaling pathway may serve as a potential target for the intervention and treatment of PM2.5-associated allergic respiratory diseases. At the public health level, these findings offer crucial scientific foundations for developing more precise environmental health policies and personalized protective strategies.

## Data Availability

The datasets presented in this study can be found in online repositories. The names of the repository/repositories and accession number(s) can be found in the article/Supplementary Material.
